# Confronting Rapid Reemergence of COVID-19 in Metropolitans of Vietnam: Updated Vietnam's Policies and Response Measures

**DOI:** 10.3389/fpubh.2022.790370

**Published:** 2022-06-16

**Authors:** Anh Minh Le, Nam Gia Dao, Carl A. Latkin, Linh Gia Vu, Vu Anh Trong Dam, Giang Thu Vu, Cyrus S. H. Ho, Roger C. M. Ho

**Affiliations:** ^1^Institute of Health Economics and Technology, Hanoi, Vietnam; ^2^Bloomberg School of Public Health, Johns Hopkins University, Baltimore, MD, United States; ^3^Institute for Global Health Innovations, Duy Tan University, Da Nang, Vietnam; ^4^Faculty of Medicine, Duy Tan University, Da Nang, Vietnam; ^5^Center of Excellence in Evidence-Based Medicine, Nguyen Tat Thanh University, Ho Chi Minh City, Vietnam; ^6^Department of Psychological Medicine, Yong Loo Lin School of Medicine, National University of Singapore, Singapore, Singapore; ^7^Institute for Health Innovation and Technology (iHealthtech), National University of Singapore, Singapore, Singapore

**Keywords:** COVID-19, response, policy, measures, collaboration

At the beginning of the COVID-19 pandemic, Vietnam was considered one of the most vulnerable countries to the pandemic crisis due to its long border with other countries, especially China, dense population, and limited medical infrastructure. Yet, with a national response plan consisting of strict anti-epidemic measures being timely introduced and rigorous early implementation, Vietnam has managed to keep the number of cases at just over 1,500 among a population of ~100 million people at the end of 2020, second among the countries with the most effective response to the pandemic (1).

As new strains of the virus [including Delta variant B.1.617 and Alpha variant B.1.1.7 with 50–70% higher transmission capacity compared to the original strain (2)] continue to emerge and threaten even the countries with a high percentage of people being vaccinated, the challenges posed for Vietnam as the fourth wave of COVID-19 are much greater than before. Instead of mostly isolated cases as in 2020, the current reemergence of COVID-19 involves cases escalating in number at a pace never seen before, forming large clusters that spread quickly from district to district, then city to city. One of the main case clusters in the current wave of COVID-19 in Vietnam is found in industrial zones, where workers crowded in poorly ventilated factories, canteens, and living quarters near their factories. The rapid level of spread is demonstrated at Hosiden Bac Giang Company, with nearly 1,000 infected workers out of 4,800 (3). Another rapidly expanding cluster of cases involves a religious group meeting that draws a large number of participants, resulting in an outbreak that spread quickly into the community, resulting in a partial lockdown of Ho Chi Minh City and a number of provinces. These clusters pose a significant challenge to the government in terms of activity control, tracing, zoning, and isolating work. With nearly 65% of COVID-19 identified cases in the fourth wave have no clinical symptoms in the first 10 days (10), it is difficult to detect positive cases based on screening, making it more likely to occur outbreaks in the surrounding community (10).

Vietnam has made major adjustments across a variety of sectors in response to the fourth wave, as the population and virus strains have changed. Table 1 outlines and compares the variations in each reemergence's characteristics and policies implemented by Vietnam.

**Table 1:** The variations in each COVID-19 reemergence's characteristics and policies implemented by Vietnam.

**Period**	**1**	**2**	**3**	**4**
Breakout date	1/23/20	7/25/20	1/27/21	4/27/21
The site of the outbreak	Ho Chi Minh City	Da Nang	Hai Duong	Many localities
Spread level	19 provinces and cities, including 3 big cities: Hanoi, Da Nang, and Ho Chi Minh City.	9 provinces and cities, including 3 big cities: Hanoi, Da Nang, and Ho Chi Minh City.	13 provinces and cities, including 3 big cities: Hanoi, Hai Phong, and Ho Chi Minh City.	62 provinces and cities reported cases nationwide, except Cao Bang.
Number of infections	415	1,133	1,302	Averagely 8.000 cases detected/ day
Quarantine policy	- Take a single test sample. - Centralized isolation for F0 and F1 (F0 is referred to as a positive case, F1 is referred to as an individual who has direct contact with F0). - Self-quarantine and health monitoring at home for F2 and F3.	- Allow 5 samples to be pooled in one test. - Centralized isolation for F0 and F1. - Self-quarantine and health monitoring at home for F2 and F3.	- Allow 10–16 samples to be pooled in one test. - Isolation: Children under 6 years old were isolated at home. Children above the age of six were isolated at the center for the first seven days and tested on days 1, 3, and 7. They were self-quarantined at home if the results were negative. The remainder of the subjects were isolated for 14 days. - During transportation, all cargo drivers and accompanying passengers were tested every two days and adhere to epidemic prevention and control methods.	- Allow 10–16 samples to be pooled in one test. - Extend the period of concentrated isolation from 14 to 21 days from 5/5/2021(4). - Centralized isolation for F0 and F1. - Self-quarantine and health monitoring at home for F2 and F3.
Treatment policy	Concentrated	Concentrated	Concentrated	- On 7/21/21, Ho Chi Minh City directed F0 to treat at home if the RT-PCR result is >30 and the patient meets the Ministry of Health's criteria. - On 8/26/2021, the Ministry of Health issued the COVID-19 treatment policy using the three-block pyramid to avoid overcrowding at hospitals. The first block isolates and treats suspected infected, asymptomatic infected, and mildly unwell individuals, while the second and third blocks treat moderate and severe cases, respectively (5).
Social support policy	- Businesses whose employees were suspended for a period of 3 months are eligible for a zero-interest loan. - Financial assistance to unemployed individuals, low-income and near-poor households (6).	- Businesses whose employees were suspended for a period of 3 months are eligible for a zero-interest loan. - Financial assistance to unemployed individuals, low-income and near-poor households.	- Businesses whose employees were suspended for a period of 3 months are eligible for a zero-interest loan. - Financial assistance to unemployed individuals, low-income and near-poor households.	- Assistance to small enterprises and unemployed workers. - Reduce employee taxes, electricity, rent, and social insurance. - Loans to firms to cover employee salaries (7).
Anti-epidemic measures	Airport temperature checks, social distancing, temporary shut down schools, non-essential business services, and cancel public events.	Airport temperature checks, social distancing, temporary shut down schools, non-essential business services, and cancel public events.	- Strict adherence to the 5K message, including *khau trang* (face masks), *khu khuan* (disinfection), *khong tu tap* (no gatherings), *khoang cach* (distancing), and *khai bao y te (*health declaration), is mandatory (8). - Online health declaration is required. - Encouragement to download BlueZone, a smartphone application that detects the risk of COVID-19 infection.	-Strict handling of cases where people do not follow the rules in a variety of ways. + The act of concealing or failing to declare health status will be fined between $500 and $1,000. + Individuals who refuse or evade quarantine shall be subject to criminal liability, with a penalty of up to 12 years in prison (9).

Vietnam has significantly increased its testing capacity and immunization coverage during the fourth outbreak. Following the efforts during the third wave to improve testing, the Ministry of Health continues to allow 10–16 samples to be pooled in one test. It is confirmed that combining many samples in one test is completely feasible and accurate with high sensitivity and specificity (11). This method also enables health workers to test more individuals with the same amount of resources (11), allowing the Ministry of Health to promote testing at optimal costs. From April 29th to June 1st, 1,374,503 samples have been tested for a total of 2,703,103 people (12). The testing capacity at hospitals has gone up by 1.7 times compared to the peak of the second epidemic (13).

In early March, nationwide training for health workers was set up to organize the COVID-19 vaccination campaign (14). Nearly 23% of the population over the age of 18 had received at least one dose of vaccine by August 30th (15), with a normal response rate of about 30% after vaccination, lower than the report (16). According to the plan, Vietnam will vaccinate 75 million people in 2021, making it the largest vaccination campaign ever (17).

Maintaining present measures, particularly stakeholders engagement and community mobilization, are critical in the fight against COVID-19. While the military has been in charge of managing the isolation centers from the beginning of the pandemic, the interdisciplinary task force set up contacts tracing to stop community spread. The media agencies were constantly updating information about the pandemic situation to the public. Although Vietnam has only 98 million people, social platforms have sent ~20 billion warning messages about measures to prevent epidemics. It is also vital to maintain introducing social security packages in these challenging times, which had a profound impact by supporting people, enterprises, and organizations to survive, especially in the time of social isolation (18).

Disease control criteria must remain fundamental, and it is up to each locality to develop adaptation plans to the outbreak. Some provinces can continue to isolate F0 from the community, others combine quarantines in a narrow area with an increase in vaccination rates. For the COVID-19 pandemic, ministries, branches, and local governments must have explicit and detailed instructions for implementation and control (19). Moreover, strategies must be flexible and adapt to each wave of the pandemic.

## Author Contributions

AL, ND, CL, and CH: conceptualization. LV, VD, and GV: data curation. ND, LV, and VD: formal analysis. CL, CH, and RH: supervision. AL, ND, VD, CL, and RH: writing—original draft. AL, LV, GV, CH, and RH: writing—review and editing. All authors contributed to the article and approved the submitted version.

## Funding

This research was supported by Gia Lam Urban Development and Investment Company Limited, Vingroup, and supported by Vingroup Innovation Foundation (VINIF) (Grant No. VINIF.2020.COVID-19.DA03). The article process charge of this paper was supported by NUS Department of Psychological Medicine (R-177-000-100-001, R-177-000-003-001, and R177000702733) and NUS iHeathtech Other Operating Expenses (R-722-000-004-731).

## Conflict of Interest

The authors declare that the research was conducted in the absence of any commercial or financial relationships that could be construed as a potential conflict of interest.

## Publisher's Note

All claims expressed in this article are solely those of the authors and do not necessarily represent those of their affiliated organizations, or those of the publisher, the editors and the reviewers. Any product that may be evaluated in this article, or claim that may be made by its manufacturer, is not guaranteed or endorsed by the publisher.
